# HSPG-Binding Peptide Corresponding to the Exon 6a-Encoded Domain of VEGF Inhibits Tumor Growth by Blocking Angiogenesis in Murine Model

**DOI:** 10.1371/journal.pone.0009945

**Published:** 2010-04-01

**Authors:** Tong-Young Lee, Judah Folkman, Kashi Javaherian

**Affiliations:** 1 Vascular Biology Program, Department of Surgery, Children's Hospital Boston and Harvard Medical School, Boston, Massachusetts, United States of America; 2 Center of Cancer Systems Biology, Department of Medicine, St. Elizabeth's Medical Center, School of Medicine, Tufts University, Boston, Massachusetts, United States of America; Bauer Research Foundation, United States of America

## Abstract

Vascular endothelial growth factor VEGF_165_ is a critical element for development of the vascular system in physiological and pathological angiogenesis. VEGF isoforms have different affinities for heparan sulphate proteoglycan (HSPG) as well as for VEGF receptors; HSPGs are important regulators in vascular development. Therefore, inhibition of interactions between VEGF and HSPGs may prevent angiogenesis. Here, we demonstrate that an HSPG-binding synthetic peptide, corresponding to exon 6a-encoded domain of VEGF gene, has anti-angiogenic property. This 20 amino acids synthetic peptide prevents VEGF_165_ binding to several different cell types, mouse embryonic sections and inhibits endothelial cell migration, despite its absence in VEGF_165_ sequence. Our *in vivo* anti-tumor studies show that the peptide inhibits tumor growth in both mouse Lewis-Lung Carcinoma and human Liposarcoma tumor-bearing animal models. This is the first evidence that a synthetic VEGF fragment corresponding to exon 6a has functional antagonism both *in vitro* and *in vivo*. We conclude that the above HPSG binding peptide (6a-P) is a potent inhibitor of angiogenesis-dependent diseases.

## Introduction

The formation of new blood vessels from pre-existing vasculature is defined as angiogenesis. The relevance of angiogenesis is not limited to cancer, but also extends to a number of non-neoplastic diseases, including macular degeneration, psoriasis, endometriosis and arthritis [1], [2]. Remodeling of blood vessels occurs through a number of steps. Following destabilization of existing vessels, it is followed by migration of endothelial cells, and proliferation and formation of new vessels tubes. Inhibition of this process can be achieved by blocking any of these stages [3].

Pericytes and lymphocytes release a number of factors which play important roles in establishing angiogenesis. They include acidic and basic FGF, tumor necrosis factor TNF-α, transforming growth factor TGF-β, and most importantly, vascular endothelial growth factor VEGF. This growth factor exists under several isoforms, i.e. VEGF_121_, VEGF_165_, VEGF_189_ and VEGF_206_, which are generated by alternative mRNA splicing. VEGF isoforms have different affinities for heparan sulphate as well as for VEGF receptors, and play distinct roles in vascular development [4]–[6].

Mast cells secrete heparin which is a linear polysaccharide with a high sulfate content. It demonstrates pro-coagulant activity. Heparan sulfate, a closely related molecule, contains much lower sulfate and is associated with the extracellular matrix (ECM) on the cell surface of all mammalian cells. It is a component of heparan sulfate proteoglycans (HSPG) which are attached to the cell membrane. The composition of polysaccharides and their sulfation content confers structures on the cell surface which determine the binding of a number of ligands to the cell surface [4]. The linear HSPGs binding domain of VEGF is located on exon 6 [7]–[9]. However, the interaction of this domain for VEGF binding to its receptors is poorly understood.

In this study, we used a synthetic peptide, corresponding to exon 6a, to compete VEGF_165_ binding to cell surface and prevent the VEGF function both *in vitro* and *in vivo*. We found that this fragment could inhibit VEGF binding to different cell types and block VEGF-induced endothelial cell migration. Moreover, this peptide suppressed tumor growth by antiangiogenesis without causing toxicity. These findings indicate that the 6a-P is a potent angiogenic inhibitor with potential for clinical investigation.

## Results

In order to test the specificity of antiangiogenesis efficacy of HSPG binding site of VEGF, we used a 20-amino acid synthetic peptide, called 6a-P which is encoded on exon-6a domain of the human VEGF gene (Materials and Methods). The mouse version of this peptide shows a single amino acid substitution at tyrosine by phenylalanine.

### 6a-P peptide binds strongly to heparin and prevents VEGF_165_ binding to different cell types by FACS

**Figure 1:**
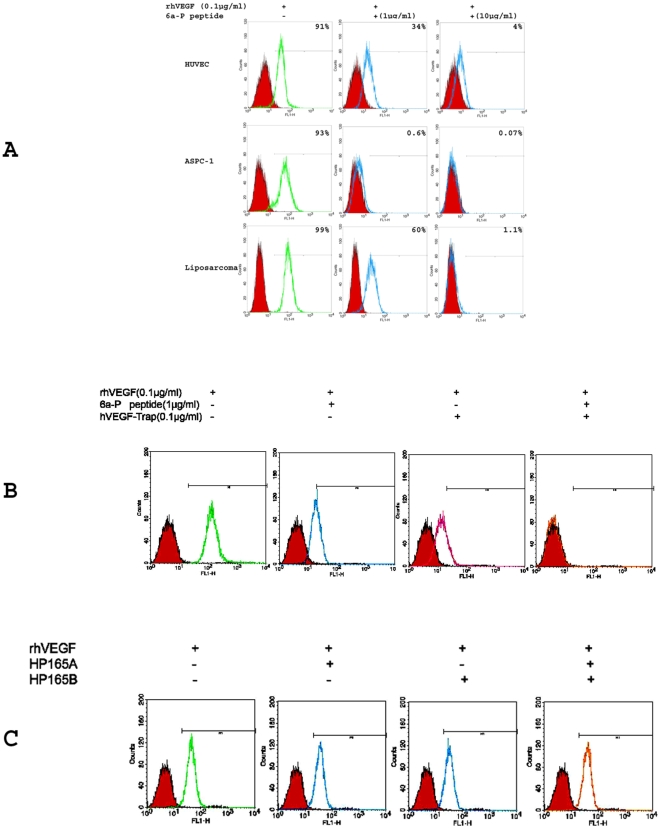
6a-P peptide prevents VEGF binding to different cell types by FACS analysis . HUVECs, fibroblasts, AsPC-1 and liposarcoma cells were subjected to FACS analysis. Cells were digested and resuspensed with 2% BSA in PBS for blocking, 0.1 µg/ml rhVEGF was incubated and present or absent with 1 (low dose) or 10 (high dose) µg/ml of the peptide for 1 h in 4°C. After cold PBS washing, the samples were incubated with VEGF antibody and secondary FITC-labeled antibody. The results show that rhVEGF binding activity is inhibited when the peptide is present (A). VEGF-Trap has been used for a positive control, inhibiting VEGF binding about 70%. The synergic result shows that the rhVEGF binding is inhibited completely when 1 µg/ml of the peptide plus 0.1 µg/ml of VEGF-Trap are present with HUVECs (B). HP165A, HP165B peptides do not inhibit binding of VEGF to HUVECs (C).

VEGF is an important angiogenesis factor, not only for endothelial cells but also tumor cells. The binding of VEGF to endothelial cell surface is mediated by HSPG. To test binding of the peptide 6a-P to proteoglycans, we employed a small heparin-sepharose column for binding studies. Most of the peptide was eluted at concentration of 1 molar NaCl as indicated in Table 1. In order to investigate the interactions between VEGF and HSPG, several cell lines such as HUVECs, liposarcoma cells, pancreatic cancer cells (ASPC-1) and mouse LLC were used for human recombinant VEGF_165_ (rhVEGF) binding by flow cytometry analysis (FACS). 0.1 or 1 µg/ml rhVEGF was used for incubation of the cells in the presence and absence of the peptide. The HSPG binding peptide blocked VEGF binding to human liposarcoma cells, ASPC-1 cells to more than 99% and VEGF binding of HUVECs to 96% at high concentration (10 µg/ml) (Fig. 1A). VEGF-Trap is a reagent which contains the neutralizing binding domains of the two VEGF receptors FLT-1 and KDR, and it is employed as an antiangiogenic therapy in ongoing clinical trials. It functions by competing with VEGF receptors [10]. It inhibits VEGF binding to HUVECs by approximately 70%. The synergic experiment showed that the VEGF binding was inhibited completely when 6a-P plus VEGF-Trap were present in binding media of HUVECs (Fig. 1B) and human liposarcoma cells (Supplemental Figure S1). We also used two synthetic linear peptides corresponding to exons 7 and 8 (cysteines were substituted by alanines), which comprise the heparin binding region of VEGF_165_ (designated HP165-A and HP165-B). The results showed that these two linear peptides were not capable of blocking VEGF binding to cells even at high concentration, pointing to requirement for maintaining three dimensional structure of VEGF165 heparin binding region (Fig. 1C).

**Table 1:** 3.5 mg of peptide 6a-P was dissolved in 1 ml of 25 mM Tris, 0.15 M NaCl, pH = 7.4.

NaCl concentration	A_280_
Flowthrough	0.02
0.15 M wash	0.05
0.5 M	0.148
1 M	0.561
2 M	0.016

It was applied to a column containing 1 ml of heparin-sephalose (GE-Healthcare). 2 ml step salt gradient (containing 25 mM Tris, pH 7.4) was employed for elution. Absorption at 280 nm reflects concentrations of the peptide.

### Immunocytochemistry data demonstrate that the 6a-P decreases VEGF_165_ binding to the cell surface

**Figure 2:**
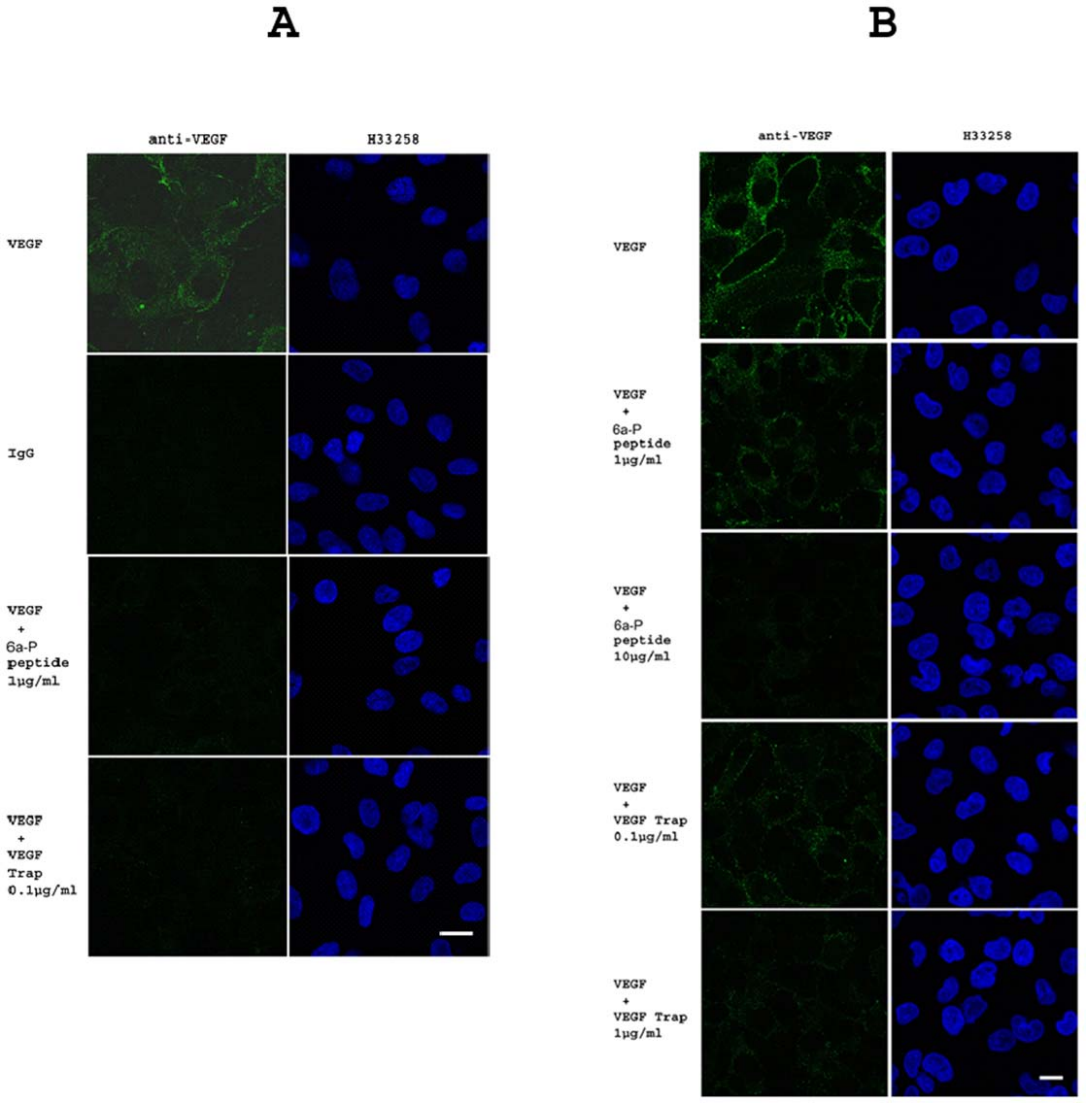
6a-P peptide decreases VEGF binding activity to cell surface by immunocytochemistry. Immunocytochemistry was used to confirm the FACS results. VEGF (0.1 µg/ml) binding intensity on HUVECS is decreased when the peptide (1 µg/ml) or VEGF-Trap (0.1 µg/ml) are present (A) (Bar, 20 µm). Similar result is shown on human liposarcoma. VEGF intensity is decreased in dose-dependent manner by 6a-P peptide (B) (Bar, 20 µm).

Immunocytochemistry experiments confirmed that VEGF binding was also inhibited by the 6a-P peptide. The VEGF binding intensity on endothelial cells (HUVECS) was decreased when the 6a-P peptide or VEGF-Trap were present (Fig. 2A). Similar results were observed with human liposarcoma cells (Fig. 2B). The VEGF intensity showed a decrease in a dose-dependent manner by the presence of the HSPG binding peptide. VEGF binding activity was also inhibited by VEGF-Trap which served as a positive control (Fig. 2B). Furthermore, *ex vivo* mouse brain embryonic sections were used to confirm this phenomenon in E14.5 mice brain. VEGF binding activity was blocked by 6a-P in embryonic mouse brain, lung and kidney (Supplemental Figure S2). These results indicate that the HSPG binding domain play an important role for VEGF interactions on the surface of the cells.

### 6a-P peptide inhibits endothelial cells migration

**Figure 3:**
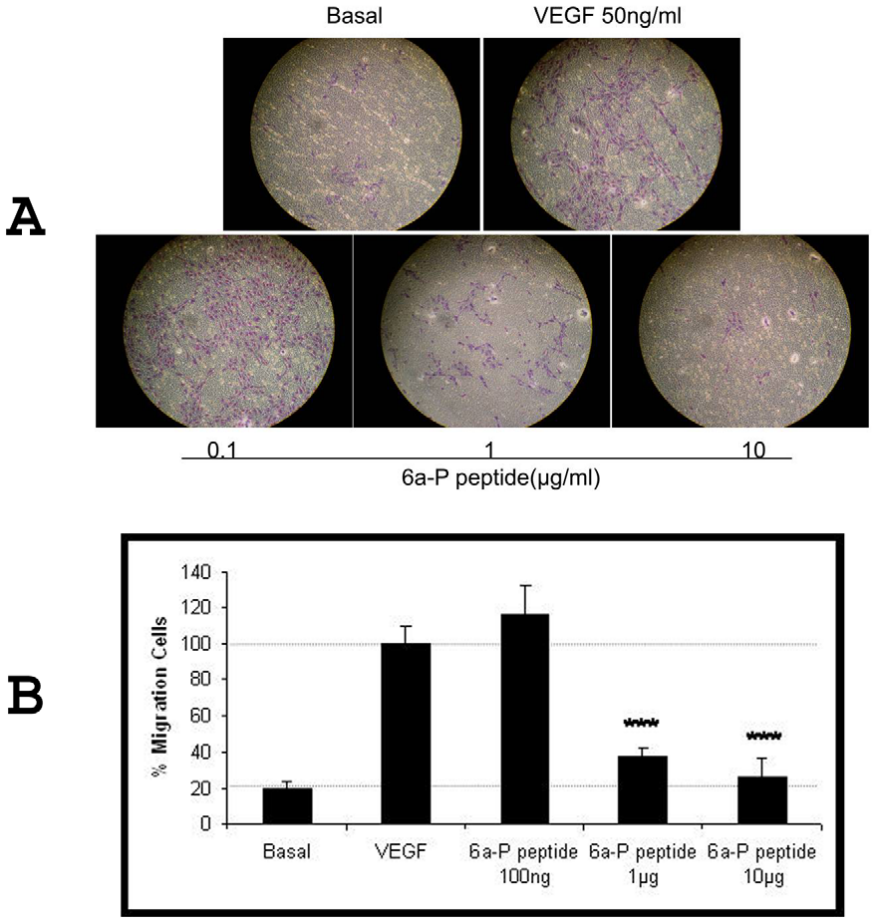
6a-P peptide inhibits endothelial cells migration. HUVECs were plated into inserts (8 µm pore size) of 24-well transwell plate and duplicated. The lower chamber was filled with serum free EBM medium containing 50 ng/ml rhVEGF plus different concentrations of the peptide for 16 h. The results show that endothelial cells migrating across the membrane are suppressed by the peptide in dose-dependent manner (A). VEGF induces endothelial cell migration is inhibited almost 90% when peptide is present at high concentration (B; P<0.001).

Endothelial cell migration is an important step in new blood vessel formation and tumor angiogenesis. To evaluate the effective of HSPG binding peptide in endothelial cell migration, we used rhVEGF_165_ to induce HUVECs migration in a transwell assay (Materials and Methods). We monitored and quantified the migration of cells. Cells migrating across the membrane were stained with blue-purple stain (Fig. 3A) and counted (Fig. 3B). The 6a-P peptide inhibited VEGF-induced endothelial cell migration at two different concentrations (P<0.001).

### 6a-P peptide inhibits angiogenesis and suppresses tumor growth *in vivo*

**Figure 4:**
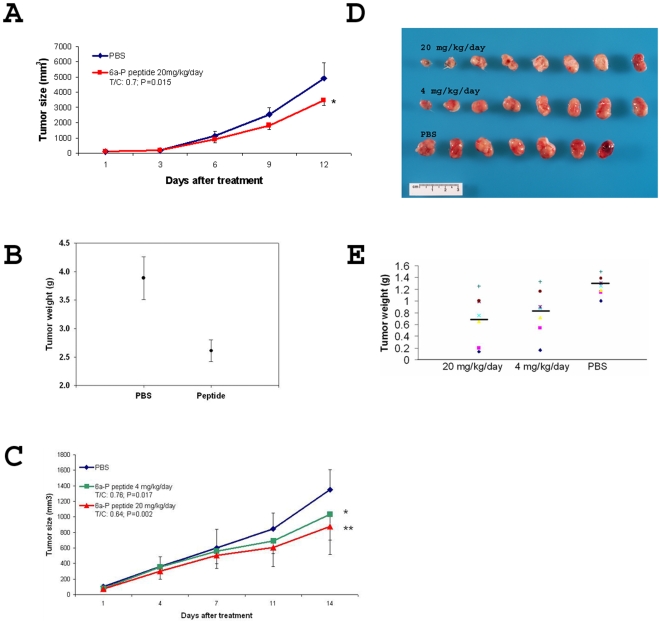
Angiogenesis and mouse LLC tumor growth are suppressed by 6a-P peptide *in vivo*. Two different tumor-bearing animal models were employed for anti-tumor study. The mice were treated with the peptide or PBS twice a day after tumor cells injection. In mouse LLC animal model, the tumor sizes are decreased about 30% after the treatments (n = 5) (A). However, the tumor weights show about 40% different between treated or non-treated groups (B). Human liposarcoma animal model shows the tumor sizes are decreased about 36% in high dose treated group (C). Less hemorrhage (as shows in red on tumor surface) is observed in the high dose of peptide treated despite similarities in tumor sizes for treated and untreated mice (D). The tumor weights show a difference of 2-fold following treatment (n = 8) (E).

Our animal experiments enable us to evaluate the efficacy of 6a-P peptide by inhibiting angiogenesis and tumor growth *in vivo*. For these studies, we used two different tumor-bearing animal models. Mice were treated with peptide or PBS twice a day. In the mouse LLC animal model, we found that tumor sizes were decreased on average about 30% at the end of treatment (Fig. 4A). However, the tumor weights showed about a 40% difference between treated and control groups (Fig. 4B). The human liposarcoma animal model showed tumor size was decreased approximately 36% in high dose treated group (Fig. 4C). Less hemorrhage (shown in red on tumor surface) was observed at the high dose of peptide treatment, even though tumor masses were in some cases similar in size to those of the control mice (Fig. 4D). The tumor weights on average were reduced 2-fold following treatment (Fig. 4E). We monitored the mouse body weights twice weekly in order to detect signs of toxicity. Peptide-treated and PBS-treated groups showed similar body weights at the end of the experiment with no significant statistical difference (data not shown).

### Tumor vessel activity and tumor cells apoptosis are affected by 6a-P peptide

**Figure 5:**
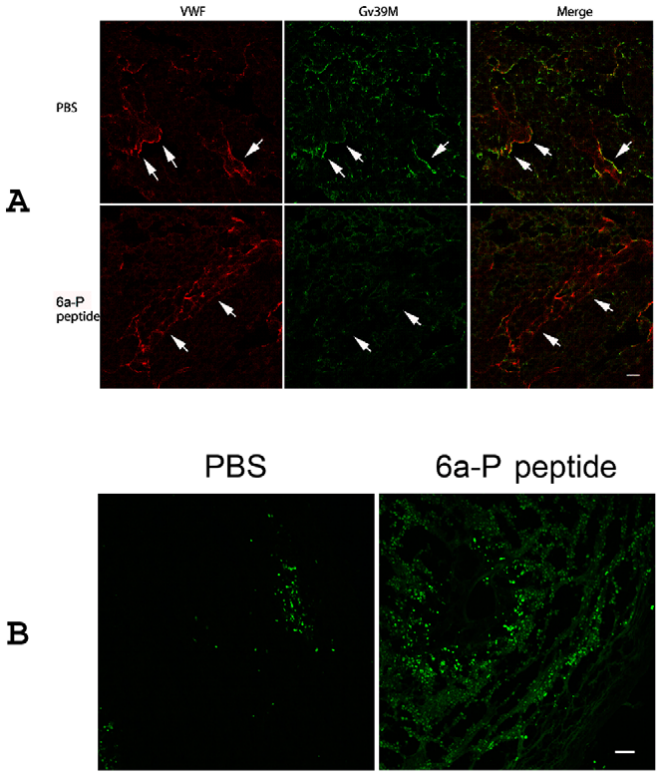
Tumor vessel activity is inhibited by 6a-P peptide. To verify the inhibition of complex of VEGF with KDR, we used the Gv39M monoclonal antibody to recognize VEGF/KDR complex in both peptide and PBS treated xenograft animal sections. Immunohistochemistry result shows that VEGF/KDR complexes are colocalized with vessels (VWF staining) on PBS treated tumor (A; up panel). However, the staining signal is decreased after peptide treatment (A; lower panel). The TUNEL assay shows more apoptotic cells in treated than untreated groups on tumor (B). (Bar, 100 µm)

Vessel activity is another important factor for angiogenesis in tumor. VEGF_165_ is secreted from cells and binds with high affinity to the two receptors on endothelial cells, FLT-1 and KDR [11]–[13]. FLT-1, KDR are almost exclusively expressed on endothelial cells [14] and appear to have different functions with respect to stimulating endothelial cell proliferation, migration, and differentiation [15], [16]. To verify the inhibition of VEGF complex with its receptor by 6a-P peptide, we used the monoclonal antibody Gv39M (Materials and Methods) to recognize VEGF/KDR complex in both peptide and PBS-treated xenograft animals. Immunohistochemistry results showed that VEGF/KDR complexes were colocalized with VWF on PBS-treated tumor (Fig. 5A: arrow on up panel). However, the staining signal was clearly decreased after peptide treatment (Fig. 5A: arrow on lower panel).

The number of apoptotic cells is additional evidence for verifying that 6a-P peptide is capable of suppressing tumor growth *in vivo*. The TUNEL assay showed more apoptotic cells in treated than non-treated group of tumors (Fig. 5B).

## Discussion

A single gene is responsible for generation of alternative splice forms of VEGF. Exons 1-5 of VEGF contain sequences which determine the protein sequences required for the recognition of its tyrosine kinase receptors, FLT-1 and KDR [17]–[21]. Exons 6 and 7 encode peptide domains which are responsible for two separate different heparin-binding regions on the cell surface. In general, different VEGF isoforms are distinguished by the presence of heparin- binding sequences belonging to one of the targeted HSPGs. VEGF_121_ does not bind to ECM due to its lack of heparin binding sequences. Exon 7 peptide is a component of VEGF_165_, whereas VEGF_189_ contains both exon 6a and exon 7. Finally, VEGF_206_ contains both exons 6a and 6b of the VEGF gene. VEGF _121_ and VEGF_165_ are secreted into the medium by producing cells, whereas VEGF_189_ and VEGF_206_ are sequestered by cell surface heparan sulfates [19], [22]–[24]. However, the contribution of exon 6a to the biological properties of VEGF has not been studied in detail.

In this report, we investigated a synthetic peptide corresponding to the exon 6a-encoded domain of VEGF gene, which is known as an ECM or heparin-binding region. We found that the VEGF_165_ binding to cell surface was blocked by this peptide in a competitive binding study by FACS (Fig. 1) and immunocytochemistry (Fig.2). The functional analysis showed that it inhibited endothelial cell migration in a dose-dependent manner (Fig. 3). For comparison purposes, we employed two synthetic peptides corresponding to exons 7 and 8, which comprise the heparin binding region of VEGF_165_ (designated HP165-A and HP165-B). This region contains several disulfides which are probably important for the heparin binding properties of VEGF_165_ [25]. In order to confer solubility to the peptides, we replaced the cysteins by alanines. The results showed that the two modified peptides were not capable of blocking VEGF binding to cells even at high concentration (Fig. 1C), pointing to importance of secondary structure and formations of proper disulfides for such an interaction.

Our animal experiments demonstrated that the tumor masses had fewer blood vessels present on tumor surfaces after low or high concentrations of the peptide treatment (Fig. 4D). VEGF and its receptors are upregulated under hypoxic conditions that are found in most solid tumors. Up-regulation of both the ligand and its receptor, specifically in tumor sections, leads to a high concentration of VEGF:receptor complex on tumor endothelium, as compared with the endothelium in normal tissue [11]. Our results showed that the interaction of VEGF and its receptor, KDR, was suppressed when this peptide was present (Fig. 5A).

Proteoglycans are associated with the mammalian cell membrane. Their genetics have been studied in detail in *Drosophila melanogaster* and *Caenorhabditis elegans*. Gradient formation and signal trasnductions are to a great extent modulated by HSPGs [6], [26]–[28]. Several prominent signaling cascades are disrupted by mutations in these acidic glycoproteins [29]–[31]. In support of this possibility, our HSPG binding peptide treated tumor section showed an increase in apoptotic cells (Fig. 5B). It may explain the phenomenon of angiogenic and tumor growth suppression observed in animals treated with exogenous 6a-P peptide, resulting in competition with endogenous VEGF binding to its targets. Our peptide, although based on its sequence, appears to be of a more non-specific nature, it is capable of competing with binding between VEGF_165_ and its heparin binding domain. We found that the 6a-P peptide could not only block VEGF binding and inhibit its function on the cells, but also suppress tumor growth and angiogenesis in animal models. These results serve as evidence that HSPG domain of exon 6a may play an important role in regulating VEGF function and its signaling transduction pathway.

In summary, we showed in this study that 6a-P peptide corresponding to exon 6a inhibited, in a concentration-dependent manner, the binding of VEGF_165_ to endothelial cells and tumor cells and VEGF-induced endothelial cell migration. It also blocked angiogenesis in the matrigel assay and suppressed tumor growth in tumor-bearing mice models. Moreover, we have demonstrated that VEGF/KDR complex formation was decreased and the apoptotic cells were increased after peptide treatment. This VEGF inhibitor may be a potential lead compound for the development of therapeutic agents against tumor angiogenesis or angiogenesis-dependent diseases.

## Materials and Methods

### Cell lines and materials

The cell lines: human Liposarcoma (a gift from Dr. George Naumov), human pancreatic cancer ASPC-1 (ATCC), mouse LLC (ATCC) were cultured in DMEM or RPMI 1640 with L-glutamine, respectively, and supplemented with 10% FCS and antibiotics. HUVECs (Lonza) were maintained in EBM endothelial growth media and EGM Bullet Kit (Lonza) with antibiotics. VEGF-Trap was a gift from Regeneron Pharmaceuticals Corporation (Tarrytown, NY). The 6a-P peptide “KSVRGKGKGQKRKRKKSRYK” and the two truncated VEGF_165_ heparin-binding peptides, HP165A “RRKHLFVQDPQTAKCSAKNTD-SRAKAR” and HP165B “KARQLELNERTARADKPRR” were synthesized by Synpep Corp. (Dublin, CA)

### Flow-cytometry analysis of VEGF binding on cell-surface

Different cells were trypsinized and resuspended in cold 2% BSA in PBS for 30 min at 4°C, and incubated with 0.1∼1 µg/ml recombinant human VEGF (rhVEGF) (R&D). After cold PBS washing, the samples were incubated with Avastin (an antibody against VEGF produced by Genentech) followed by the secondary FITC-labeled antibodies (Sigma) and analyzed by BD Biosciences FACS Calibur flow cytometer.

### Immunocytochemistry

The *in vitro* staining of HUVECs and Liposarcoma cells were plated on cover slips or the *ex vivo* staining of mouse embryonic frozen section E14.5 slides were fixed by 4% paraformaldehyde for 10 min. Cells or sections were incubated with 100 ng/ml of rhVEGF present or absent with 1 or 10 µg/ml synthetic heparin-binding peptide for 1 h. After PBS washed, the slides were detected by Avastin and Alexa 488-labeled IgG. The slides were imaged by confocal-microscopy (model DM IRE2; Leica). DAPI or Hoechst33258 counterstaining of nuclei are shown in blue.

### Endothelial cell migration assay

HUVECs were washing by serum free EBM medium twice and re-suspended containing 5×10^4^ cells/well in 0.6 ml with medium were plated into 24-well inserts (Coring, 8 µm pore size) in duplicatation. The lower chamber was filled with 0.6 ml of serum free EBM medium containing 50 ng/ml rhVEGF (R&D) plus different concentrations of 6a-P peptide. After incubation for 16 h at 37°C, the cells were fixed by methanol and stained with eosin and hemotoxlin. Cells on the upper side of the transwell membrane were removed by cotton swab. Cells migrating to the lower side of membrane were counted.

### Animal and tumor models

All animal procedures were carried out in compliance with Children's Hospital Boston guidelines. Protocols were approved by the Institutional Animal Care and Use Committee. Eight weeks old male and pregnancy female C57B/6J mice (The Jackson Laboratory) and SCID mice (Massachusetts General Hospital) were used. Mice were acclimated, caged in groups of five in a barrier care facility, and fed animal chow and water ad libitum and all mice were shaved and the dorsal skin was cleaned with ethanol before cells injection. Human liposarcoma or mouse LLC cells were used for anti-tumor studies. A suspension of 5×10^6^ of liposarcoma or 1×10^6^ of LLC cells in 0.1 ml of PBS was injected. Mice were weighed and tumors were monitored twice a week in two diameters with digital calipers. Tumor volumes were determined using a^2^×b×0.52 (where a is the shortest and b is the longest diameter). Tumors were allowed to grow to ∼100 mm^3^ and mice were randomized. The treatments were by bolus s.c. injections twice a day.

### Immunohistochemistry and TUNEL assay

Tumors, organs and E14.5 embryo were embedded in OCT medium (EMS). Sections were rinsed by cold PBS and fixed with 4% paraformaldehyde for 10 min before staining. Apoptosis was examined by use of the terminal deoxynucleotidyltransferase-mediated deoxyuridine triphosphate nick end labeling (TUNEL) assay [32] through the manufacturer's protocol (Promega). Antibody von Willebrand Factor (Dako) was used for vessels staining. Gv39M (a gift from Dr. Philip E. Thorpe) was used for VEGF:KDR complex staining [33]. The primary antibodies were detected by Alexa 488 or 594-labeled secondary antibodies (Molecular Probes). The sections were imaged by confocal-microscopy (model DM IRE2; Leica). For tumor vessel density, VWF positive staining was counted in five fields by pixels number. Data are expressed as mean ±SD.

### Statistical method

Data are expressed as mean ± SD. We considered a P value below 0.05 as significant by using two-sided unpaired Student's *t* test.

## Supporting Information

Figure S1 (0.25 MB TIF)

Figure S2 (2.41 MB TIF)
