# Supplementary figures and images for: HSPG-Binding Peptide Corresponding to the Exon 6a-Encoded Domain of VEGF Inhibits Tumor Growth by Blocking Angiogenesis in Murine Model
Source: PLoS One. 2010 Apr 1;5(4):e9945. doi: 10.1371/journal.pone.0009945 (PMC2848586; doi:10.1371/journal.pone.0009945)

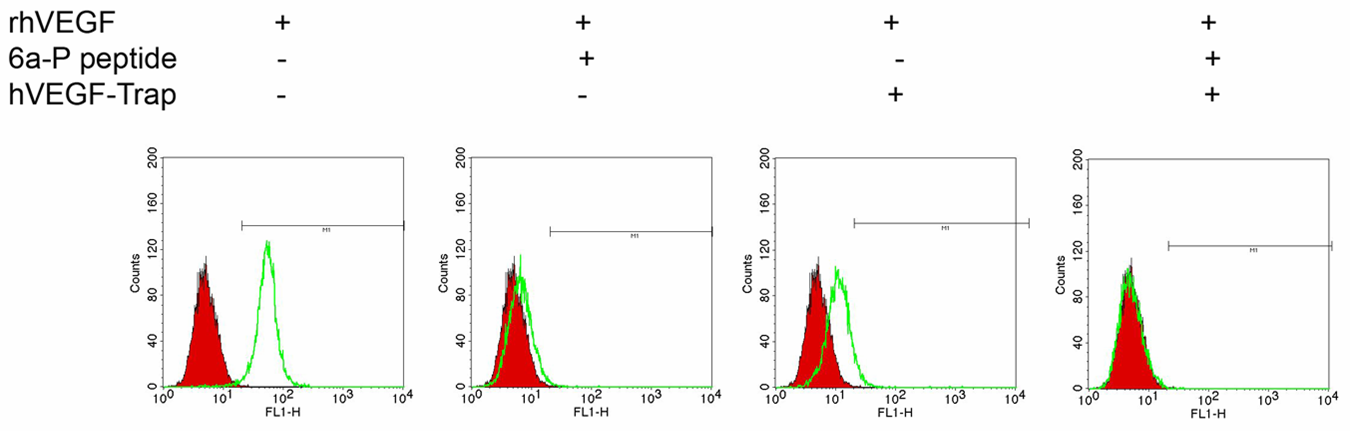

Supplement: Figure S1 — (0.25 MB TIF) [file pone.0009945.s001.tif]

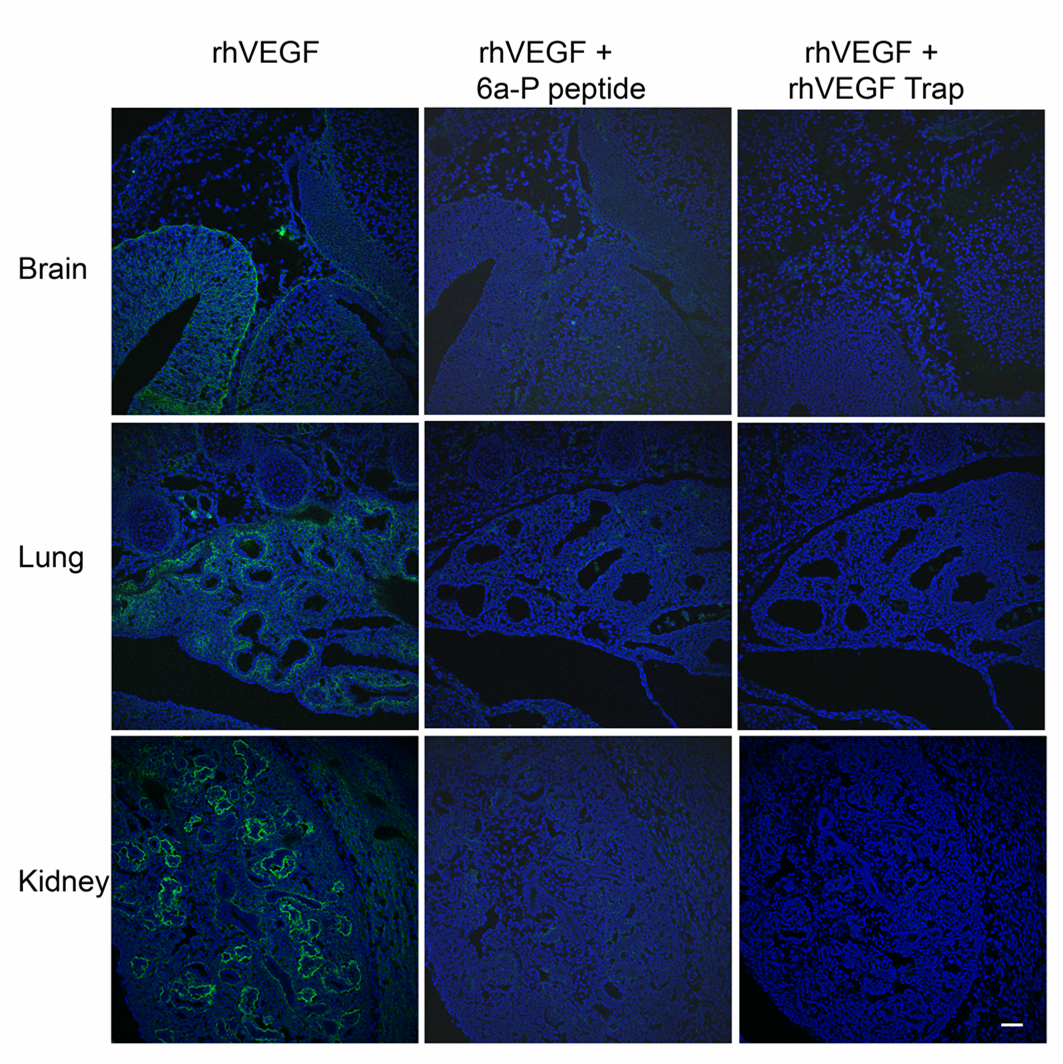

Supplement: Figure S2 — (2.41 MB TIF) [file pone.0009945.s002.tif]
